# Influence of Academic Performance, Level of Play, Sports Success, and Position of Play on the Motivation of the Young Football Player

**DOI:** 10.3390/ijerph17103374

**Published:** 2020-05-12

**Authors:** Christian Ureña-Lopera, Honorato Morente-Oria, José Luis Chinchilla-Minguet, Alfonso Castillo-Rodríguez

**Affiliations:** 1 Department of Physical Education and Sports, University of Granada, 18010 Granada, Spain; christian.urena.lopera@gmail.com; 2 Department of Languages, Arts and Sports, University of Málaga, 29079 Málaga, Spain; hono@uma.es (H.M.-O.); jlchinchilla@uma.es (J.L.C.-M.)

**Keywords:** intrinsic motivation, extrinsic motivation, demotivation, soccer, competition, training

## Abstract

Background: Motivation in athletes is a state that fluctuates due to multiple factors that can, in turn, negatively or positively influence sports performance. Objectives: The aim of this study was twofold, being, on the one hand, to analyze the motivation of soccer players of developmental age in two different contexts (training time (baseline) and the precompetitive time) depending on the category, sports success and playing position, and, on the other hand, to find relations of the motivation dimensions with the academic performance and physical characteristics of the soccer players. Methods: One hundred and forty-one under 16 (U16) soccer players were selected (age: 14.7 ± 0.5; height: 170.4 ± 7.2 cm; weight: 61.6 ± 10.0 kg). Data on academic performance, physical and socio-demographic characteristics were recorded, and in two differentiated moments, the motivation dimensions, both in training and in competition. Results: The results showed that the general motivation decreases with the competition, and in particular, the intrinsic motivation, where the precompetitive evaluation is lower than the basal, in both categories (*p* < 0.05). In addition, demotivation is explained by 10.2%, 19.8%, and 23.9% by fat mass, by academic performance, and by category, respectively; and the extrinsic motivation of external regulation is explained in 26.0% by the academic performance factor (*p* < 0.01). Conclusions: U16 soccer players show lower levels of motivation at moments prior to the sports competition, and these dimensions of motivation are explained by the category, academic performance, and fat mass.

## 1. Introduction

Around 265 million people play soccer in the world, being more than half under 18 players (U-18) [1], a large part of them taking part in organized sports competition [2]. This high influx and popularity lead to the existence of a high injury rate [3], the participation of low-qualified trainers [4], high levels of wastage [5], but mainly, excessive emphasis on early sports specialization [6]. Many coaches aim to find the best performance for the team and the players, but among the factors that make up this maximum performance is the psychological and emotional aspect of the player [7,8]. Athletes in early adolescence produce a great social change, the result of morphological, hormonal, physiological change, which has the consequence of more aggressive and non-pro-social behaviors in general, which has its immediate consequence in the family and in close friends [9]. For these reasons, adherence in sports practice at these ages is presented as an arduous task for coaches.

According to the Sports Participation Development Model, there are two potential ways to reach the elite; on the one hand, an early specialization which involves practicing a primary sport almost exclusively, and on the other hand, an early multi-sport practice, where in the first years of sports practice, young people will participate in multiple sports [10]. This second path is the one that best seems to lead to the real objective of permanence in sports practice, since it will be the one that makes young athletes experience different physical, cognitive, affective, and social environments [11], and having greater wealth and baggage of sports experiences, they will be able to freely choose, in the future, and without pressure, the one in which they would deliberately specialize and practice in later stages. Cox [12] postulates that people with intrinsic motivation (IM) participate in activities of their own choosing (in a self-determined way), and in addition, early multi-sport practice feeds IM [10]. Likewise, athletes who at an early age make use of this early multi-sport practice, benefit from the transfer of physical and cognitive abilities [13], finding benefits of conditional qualities such as strength, speed, endurance, and coordinative qualities in later adolescence [14]. In summary, early specialization can weaken the young athlete’s IM, and therefore also their self-determination [15], and could generate extrinsic motivation because of specialization [16]. A recent study found that in the sample of young Chinese soccer players, extrinsic and intrinsic factors had a similar impact on motivation [17].

Team sports, such as soccer, have a volitional, collective, open, and dynamic environment character, and as a consequence, enormous importance is given to cognitive skills, even at the same level as technical and tactical performance [18], and anxiety may fluctuate during training (TR) and competition (CM) [19]. Competitive anxiety is a factor to be considered in sports such as soccer, since most of the existing studies indicate the negative role it plays in performance, as well as on fun in sports, even increasing of the possibility of sports abandonment [20].

Considering the effect of CM on the players’ anxiety, it would be necessary to become aware of the need to develop and apply strategies through the TR, so that young athletes are allowed to control the different situations and emotions that somatic or cognitive anxiety could cause [21]. It is in this aspect that enhancing IM in young people could help alleviate stress and anxiety, since IM is presented as a moderator of the relationship between anxiety and academic performance [22]. Additionally, encouraging coaches to focus the motivational climate on the task during TR could thus also enhance IM as a moderating agent of competitive anxiety, since motivational climates perceived as mastery or task have been associated with low anxiety during competition [23]. Taken together, these conditioning factors lead us to hypothesize that soccer players in developing age show greater susceptibility to sports CM [8], fluctuating their baseline psychological states, to the detriment of positive psychological characteristics such as attention, concentration, self-confidence, self-esteem, among others. A coach should consider (with the intention of preparing his players for competition) the different learning rhythms, using individualized teaching styles [24], designing tasks with clear guidelines and goals [25], which pose challenges commensurate with the level and experience of their footballers, with adequate working time, and feedback where the process prevails over the result [26].

The activation of athletes during TR can be determined by an optimal psychological state, to optimize performance. Motivation is a widely studied field in this sense, which is based on various theories such as the Achievement Goals theory [27] and Self-determination theory [28]. For a trainer, knowing the motivational orientations of their practitioners is fundamental when it comes to designing tasks for TRs. The athlete must be understood as a multidimensional being, for which, in the search for sports performance, physical, technical, or tactical preparation should not prevail over psychological parameters, since it is the latter that will allow proper motor optimization and a predisposition towards sports success. In this way, athletes and trainers seek to optimize sports performance through physical condition, technical–tactical aspects, nutritional variables, and thanks to the control of psychosocial responses, among others [29].

On the other hand, IM has been shown to positively affect academic performance, learning, and achievement [30,31]. Students with high academic motivation immersed in learning are generally more likely to achieve better grades, as well as lower dropout rates [32,33,34]. Achievement motivation positively influences academic performance [35]. We find in the literature some studies that address the relationship between motivation and general academic performance [36,37]. Gutiérrez and López [38] analyzed motivational factors, behavior, and performance in their study, concluding that the best predictor of academic performance is the assessment that teachers make of student behavior. However, there are few studies carried out in the Spanish context that relate motivational factors and academic results. Likewise, there are studies that have related academic performance with physical characteristics [39,40].

However, this academic performance may not have the same effect on student motivation. For example, some studies showed a lower range of academic performance by students with higher extrinsic motivation (EM), while those with higher IM had better academic achievement results [32]. In addition, young people with demotivation showed little results in academic performance [34], appreciating a negative relationship between anxiety and academic performance, since students who experienced high levels of anxiety obtained worse results on their exams, as well as worse overall academic performance [41]. In this sense, anxiety has been considered as a negative factor of academic performance since it could also provoke in students not only psychological symptoms, such as nerves before entering class, tension during exams, or even inability to perform some tasks, but also physical symptoms such as excessive sweating, tachycardia, lowered defenses, hyperventilation, or abdominal pain [42,43].

The objectives of the present study are, first of all, to analyze the motivation of soccer players in developing age in two different contexts (moment of TR (basal) and precompetitive moment) according to the category, the sporting success and the game position; and, secondly, to find relationships of the dimensions of motivation with the academic performance and physical characteristics of soccer players.

## 2. Materials and Methods

### 2.1. Subjects

A total of 145 subjects under 16 (U16), between 14 and 16 years (mean: 14.73 ± 0.5 year-olds), who take part weekly in the official football club participated in the study. As inclusion criteria for the selection of the sample, it was considered that the players must be between 14 and 16 years old, not suffer any pathology that may alter the results in the psychosocial field, and have not been the object of serious physical injuries in the last 6 months.

### 2.2. Instruments

In order to assess the motivation of the players, we used the Spanish version of the Sport Motivation Scale (SMS) [44,45], a total of 28 items in which players are asked about the reasons that lead them to practice their favorite sport (in this case soccer), in a TR and in a CM. The answers are formulated on a Likert-type scale in which each item has a response range from 1 to 7. The score of 1 corresponds to “Never”, and the score of 7 to “Always”, with respect to the formulation of the question. This scale is made up of seven subscales of four items each, so that they evaluate the three types of IM, such as IM to knowledge (items 2, 4, 23, and 27, e.g., “I knew that my ability would allow me face the challenge that was presented to me ”), IM to achievement (items 8, 12, 15, and 20, e.g., “Because I feel very satisfied when I can adequately perform difficult RT techniques”), and IM to stimulating experiences (items 1, 13, 18, and 25, e.g., “For the pleasure of living stimulating experiences”), the three types of ME, which are external regulation (items 6, 10, 16, and 22, e.g., “Because it allows me to be well considered by the people I know), introjected regulation (items 9, 14, 21, and 26, e.g., “Because it is absolutely necessary to practice sport to be fit”), identified regulation (items 7, 11, 17, and 24, e.g., “Because, in my opinion, it is one of the best ways to meet people”), and finally, that of demotivation, (items: 3, 5, 19, and 28, e.g., “I used to have good reasons to practice it, but now I wonder if I should continue doing it”). The reliability index of the scale is 0.74 [35]. In the present study, the reliability of the instrument obtained an identical mean of α = 0.74, with the following values: IM at knowledge, α = 0.70; MI at achievement, α = 0.82; MI to stimulating experiences, α = 0.63; external regulation, α = 0.74; introjected regulation, α = 0.80; regulation identified, α = 0.81; and demotivation α = 0.67.

In addition, an ad hoc questionnaire with socio-demographic and body-type variables related to the soccer players was devised, to be completed dichotomously or freely according to the case (weight, height, age, playing position, team, years of federation (experience), hours of TR, previous injuries, and presence of relatives in the matches). The independent playing position variable was unified into four positions, following a modification of the initial classification of some studies [46,47]: goalkeepers, defenders, midfielders, and forwards. Regarding academic performance, the grades of three subjects with the highest number of hours per week, such as Mathematics, Spanish Language and Literature, and English, was taken into account. In addition, an average of the three grades was made to obtain an average grade of the student. In this way, the academic performance was divided into four parts.

### 2.3. Procedures

Various sports clubs in Malaga’s province were visited to access the sample. Appropriate permits were requested, and management, coaching staff, players, parents, and guardians were informed in detail about the objectives of the study and the treatment to be carried out of the data collected. The type of study carried out was of a descriptive and inferential cross-sectional nature, lasting one year (in the 2016–2017 season), which was carried out in three phases with data extraction at different times. The first of them, basal type, was taken during one TR session, and players filled out the sports motivation questionnaires and the ad-hoc socio-demographic questionnaire at least 48 h in advance and 48 h after the official CM [48,49]. The second moment, precompetitive character, was carried out 24 h before the official CM and included only the sports motivation questionnaire; and finally, in the third moment, the academic performance of the participants was collected, corresponding to the grades obtained in the subjects taken at their educational center.

To carry out this study, permission was requested to collect data from both the clubs and the parents of the participants, who signed the voluntary informed consent. They were submitted to the questionnaires, respecting at all times the indications established by the Declaration of Helsinki (2013) on human research. This study was approved by the Ethics Committee of the University of Granada (471 / CEIH / 2018).

### 2.4. Statistical Analyses

The SPSS statistical software package for Windows v.22 (IBM SPSS Statistics, Chicago, IL, USA) and Microsoft Office Excel (Microsoft Corp., Redmond, WA, USA) were used. Firstly, the normality test (Kolmogorov–Smirnov test) was carried out, resulting that all variables follow a normal distribution, except the academic performance metrics. Besides, the α value was calculated to find the reliability of the motivation test in the study sample. Subsequently, the statistical analysis was performed using descriptive and contrast statistics for repeated measures comparison, through the Student’s *t*-test for related samples and the analysis of variance test (ANOVA) with the game position factor, previously observing the homogeneity test (Levene’s test) and using a post hoc Bonferroni test. The threshold values for the effect size statistics were, in Student’s *t*-test and ANOVA test, small, 0.20 and 0.10; moderate, 0.50 and 0.25; and large 0.80 and 0.40, respectively [50]. Finally, relationships between motivation variables with academic performance, physical characteristics, and weekly TR session volume were analyzed (Pearson’s r and linear regressions with the stepwise mode). A significance level of *p* < 0.05 was established.

## 3. Results

Table 1 shows the differences in baseline and precompetitive motivation depending on the level of play or expertise. In general, all players showed lower motivation values in a competition context. Particularly with IM to knowledge (in C1, 22.7 ± 4.5 and 20.9 ± 4.6, *p* = 0.04), IM to stimulating experiences (in C1, 23.9 ± 3.6 and 21.5 ± 3.8, *p* = 0.002; in C2, 24.7 ± 3.0 and 20.7 ± 3.5, *p* = 0.000), demotivation (total sample: 10.7 ± 5.5 and 13.2 ± 6.1, *p* = 0.02), and general IM (in C1, 69.7 ± 11.2 and 64.5 ± 11.2, *p* = 0.01), there are significant differences concerning both contexts.

Table 2 presents the differences in baseline and precompetitive motivation based on sports success. Soccer players did not show a different motivation based on their sports success, however, both populations also showed lower values of motivation in the precompetitive context. Players without sports success presented significant differences in IM to knowledge (24.2 ± 2.9 and 21.0 ± 2.9, *p* = 0.05), IM to stimulating experiences (23.8 ± 3.3 and 20.7 ± 3.8, *p* = 0.000), and general IM (70.3 ± 10.9 and 63.3 ± 8.5, *p* = 0.05); and players with sports success, in the variables MI to achievement (23.4 ± 3.6 and 21.8 ± 4.7, *p* = 0.05), IM to stimulating experiences (24.2 ± 3.6 and 21.7 ± 3.7, *p* = 0.004) and general IM (69.6 ± 10.7 and 64.6 ± 11.9, *p* = 0.022).

**Figure 1:**
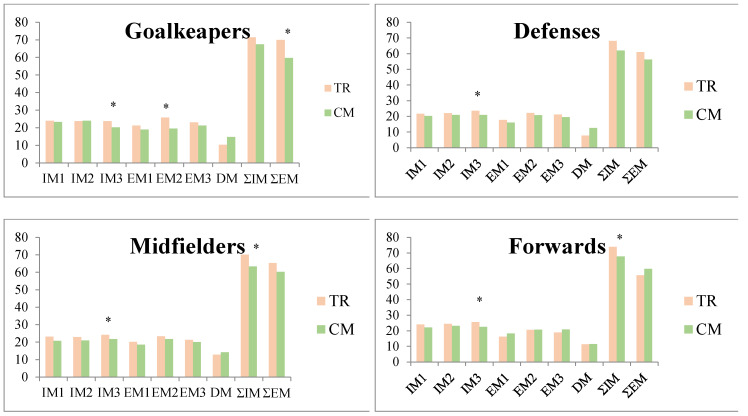
Paired *t*-test between baseline and precompetitive contexts of the motivation dimensions according to the playing position.* *p* < 0.05; TR: training; CM: competition; IM1: IM to knowledge; IM2: IM to achievement; IM3: IM to stimulating experiences; EM1: external regulation; EM2: introjected regulation; EM3: regulation identified; DM: demotivation; ƩIM: summation of all dimensions of IM; ƩEM: summation of all dimensions of EM.

Also, the players did not show differences according to their playing position, although in each one of them, a greater motivation was also shown in baseline moments of TR (Figure 1), significantly in the dimensions of IM to the stimulating experiences, in all playing positions; introjected regulation and general EM, in goalkeepers; demotivation, in defenses; and general IM, in midfielders and forwards.

On the other hand, correlation tests (Spearman’s Rho) were performed. Table 3 shows the correlations of the motivation dimensions with academic performance. In the context of TR and CM, the correlations found are negative in all cases. In TR, the correlations of the external regulation dimension and general EM with the mathematical performance, language, and general academic performance stand out (rho = −0.44 to −0.55; *p* < 0.01). In CM, the lack of motivation is correlated with the performance in language, English, and general academic performance (rho = −0.63, −0.40, −0.45; *p* < 0.05; respectively). Regarding the correlations of physical characteristics, a positive correlation is observed in TR between MI at achievement and height (r = 0.33; *p* < 0.01). In the precompetitive context, correlation of MI to achievement with weight and height is observed (r = 0.46 and 0.43; *p* < 0.01; respectively), MI to stimulating experiences and introjected regulation with height (r = 0.35 and 0.42; *p* < 0.05; respectively), and the demotivation shows a positive correlation with the percentage of fat mass and an inverse correlation with the number of weekly TR (r = 0.36 and 0.38; *p* < 0.05; respectively).

Finally, linear regressions (stepwise) were performed to check what factors can predict the dimensions of motivation. In the CM context, the demotivation dimension is predicted by fat mass (model 1) by 10.2% (Standard Error of Estimate (SEE) = 3857; *p* = 0.050); by the general academic performance (model 2) in 19.8% (SEE = 5.526; *p* = 0.012); and by category and fat mass (model 3) by 23.9% (SEE = 5.415; *p* = 0.006). In the TR context, the external regulation dimension is explained in 26.0% by the general academic performance factor (model 4; SEE = 6.013; *p* = 0.003).

## 4. Discussion

The objectives of the present study were, first of all, to analyze the motivation of soccer players in developing age in two different contexts, the TR moment (basal) and the precompetitive moment according to the category, the sport’s success and position of the game; and, secondly, to find relationships of the dimensions of motivation with the academic performance and physical characteristics of soccer players. The results showed that motivation in general decreases with CM, and in particular, MI, where the precompetitive evaluation is significantly lower than the baseline, which may be mainly due to the perception of players in sports initiation of CM as a source of stress, derived from fear of possible failure [51]. Furthermore, it is linked to greater demotivation in the precompetitive period compared with baseline, which could be caused by the direct relationships that precompetitive anxiety has been shown to have with other negative emotions, as well as inverse relationships with positive emotions [52].

The fluctuation of the IM between the basal moment and the precompetitive moment, and specifically, the IM to the knowledge and the IM to the stimulating experiences, has been evidenced in the present study. This suggests that this type of motivation decreases significantly when the CM is close, which could be due to precompetitive stress as a negative factor in this CM environment [53]. The decrease in these two types of motivation during CM could be caused by the importance attached to achieving the goal of winning (IM at achievement) rather than improving knowledge of new techniques, or even that these experiences are stimulating for the player since he can be looking for victory at any price. It also increases demotivation, which is a correlated factor with some levels of stress and that according to several authors, non-self-determined subjects, in whom EM and demotivation predominate, should not be led to such constructive commitments, which would provoke more adaptive forms during stress [54].

These negative effects of CM have been extensively studied in different psycho-physiological aspects [55,56], although there are studies that subscribe CM as a positive aspect for motivation, and these are found in the field of rehabilitation, promoting adherence to autonomous rehabilitation work at home [57]. On the other hand, a general improvement in children’s development has been found through CM as a driving vector that fosters motivation towards healthy lifestyles [55].

However, no significant differences were found in terms of motivation per specific playing position. In this sense, a recent review of studies concludes that forwards and midfielders have greater motivation, a circumstance that this study does not analyze [58]. This situation could be true since the analyzed players belonged to the U16 category, with medium levels related to sports success, and do not yet have a fully defined specific playing position and/or role in the team. Although, in all cases, the fluctuation of motivation has been perceived as a consequence of the pressure transmitted by the CM, which, as we have seen previously, directly affects the level of IM, decreasing it, and the level of demotivation, increasing it [59]. Significant differences between baseline and precompetitive measurement are found in all positions for IM to stimulating experiences. This could be since the coaches are in charge of the design of the practical sessions or TR, as well as those responsible for modifying such important variables as the grouping of athletes, or the evaluation of their performance, and all this together with their authority, creates a motivational climate that has a transcendence or direct impact on the motivation of athletes [60]. Thus, the coaches in the sample may have created task-oriented motivational climates, where enjoyment and playfulness are found only by athletes in the TR. This could explain the significant differences found between the basal moment and the precompetitive moment in IM to stimulating experiences, since this dimension of motivation acts when the individual is involved in an activity for the simple fun that it produces, or for the experimentation of the sensations that this activity makes them feel [61].

Regarding academic performance, based on our results we found only inverse correlations, standing out is the external regulation of the basal moment, which correlates in all subjects, namely, we can establish that the higher the academic performance, the less external regulation. Regarding the measurement of the precompetitive moment, we can also find an inverse correlation between academic performance and demotivation. Both cases could coincide with the opinion of Hernandez et al. [62] on the decrease in academic performance based on a physiological and psychological obstruction on the part of a submission to situations of continued stress. However, there are multiple studies on achievement motivation on school moments themselves on academic performance [35,36,37]. There is consensus on the positive relationships between the two variables, although there are no studies that address the motivation of schoolchildren about sport practiced with academic performance. When the questionnaire was provided (explained in instruments), the student was asked what motivation they have for their favorite sport, which in all cases was soccer, since it is the sport they habitually and voluntarily practice. In this sense, our study is of special scientific interest because, in addition, a sample with a medium level of expertise has been studied. There is a study that addressed the motivation of the classes and the academic performance, at ages under 18, all of them with an elite athlete’s skill level, finding strong relationships between both variables [63]. 

If we look at the results related to physical characteristics, we can see positive correlations between IM at achievement with weight and height at precompetitive times. This could be due to the important role that body composition plays in soccer [64,65]. A relationship between fat mass and demotivation is also observed, which would also corroborate the previous hypothesis that physical corpulence in terms of the optimal physical state improves IM, while higher levels of fat cause greater demotivation, which could be justified due to the high number of physical contacts that occur in each soccer match, where successive movements and skills require significant physical demand, such as kicks, short sprints, throws, collisions, changes of direction, jumps, mowing, etc. [65,66].

As a possible limitation of this study, as well as at the same time, it may serve as a suggestion for future lines of research, it would be advisable to analyze the influence of stress on more self-determined subjects (who have more IM) during CM, since they tend to face this stress by guiding it to the task, while athletes whose self-determination is lower usually guide it to disconnection in CM [54]. In the same way, it would also be interesting to carry out an analysis of the fluctuation between the motivations, between the basal moment and the precompetitive moment, of the subjects who demonstrate sporting success compared with those who do not. Finally, being able to contrast these motivational results with the player’s anxiety could help to understand the fluctuation caused at different times.

## 5. Conclusions

The main findings of this study revealed that soccer players in developing age show a lower perception of motivation in moments before sports competition, jeopardizing the monitoring of the practice of this sport. Furthermore, the dimensions of motivation were explained by the variables of academic performance, category, and fat mass of the player. Thus, it seems that multiple factors can influence the fluctuation of motivation, not only the fact of facing the competition, where it is known that anxiety is generated and this decreases motivation, but also, academic performance could influence the demotivation and extrinsic motivation by up to 19.8% and 26% of the explained variance, respectively. As practical applications, coaches, physical trainers, and professionals in general of this sport are urged to contribute to the levels of motivation through strategies and teaching methods that enable decision-making and greater prominence for the soccer player in development age, to reduce or alleviate the negative effects that the competition can have in soccer players of these ages and categories that are more vulnerable. The soccer coach should create situations similar to the CM, which, redirected through the high motivation that soccer players have in the TR context, can be extrapolated to a greater motivation during the CM. On the other hand, a good physical conditioning of the players, as well as a good diet, which enables a lower percentage of fat mass, could, as has been seen in the results, have a buffering effect on demotivation. Also, a lower weight, always seen from a healthy point of view, can increase IM. Also, use socializing teaching styles, propitiating help between classmates and creating team feeling. All of the above measures are stated with the aim of promoting greater motivation of the players in TR, and by extension, to CM.

## Figures and Tables

**Table 1:** Mean and standard deviation were calculated in baseline and precompetitive contexts with the motivation variables according to the level of play (expertise).

Motivation	Level of Play	TR ^1^ (*N* = 141)			CM (*N* = 141)			*p*
IM1	C1	22.73	±	4.48	20.92	±	4.58	0.044
	C2	22.64	±	4.87	21.57	±	4.20	0.653
	Total	23.01	±	4.53	21.17	±	4.50	0.028
IM2	C1	23.08	±	4.49	22.08	±	4.47	0.287
	C2	22.86	±	3.44	20.57	±	4.32	0.250
	Total	23.31	±	4.28	21.77	±	4.53	0.070
IM3	C1	23.87	±	3.58	21.52	±	3.79	0.002
	C2	24.71	±	2.98	20.71	±	3.45	0.000
	Total	24.27	±	3.46	21.53	±	3.75	0.000
EM1	C1	18.14	±	6.46	17.17	±	5.77	0.433
	C2	18.71	±	6.45	20.57	±	5.22	0.280
	Total	18.81	±	6.58	17.84	±	5.72	0.369
EM2	C1	22.23	±	5.16	21.08	±	4.19	0.325
	C2	22.86	±	8.67	20.71	±	4.39	0.342
	Total	22.69	±	5.89	20.96	±	4.46	0.100
EM3	C1	20.71	±	4.92	20.15	±	3.94	0.516
	C2	20.29	±	6.87	20.57	±	4.47	0.887
	Total	20.90	±	5.25	20.17	±	4.06	0.361
DM	C1	9.98	±	5.16	11.39	±	5.82	0.198
	C2	13.29	±	7.25	18.71	±	3.90	0.120
	Total	10.70	±	5.54	13.17	±	6.14	0.021
ƩIM	C1	69.67	±	11.2	64.52	±	11.2	0.013
	C2	70.21	±	8.69	62.86	±	9.86	0.098
	Total	70.60	±	10.8	64.47	±	11.1	0.001
ƩEM	C1	61.08	±	14.0	58.40	±	11.2	0.296
	C2	61.86	±	21.0	61.86	±	13.4	0.990
	Total	62.40	±	15.7	58.97	±	12.0	0.153

^1^ TR: training; CM: competition; IM1: IM to knowledge; IM2: IM to achievement; IM3: IM to stimulating experiences; EM1: external regulation; EM2: introjected regulation; EM3: regulation identified; ƩIM: summation of all dimensions of IM; ƩEM: summation of all dimensions of EM; DM: demotivation; C1: category 1; C2: category 2.

**Table 2:** Mean and standard deviation were calculated in baseline and precompetitive contexts with the motivation dimensions according to the sports success.

Motivation	Sports Success	TR ^1^ (*N* = 141)			CM (*N* = 141)			*p*
IM1	S	21.98	±	5.00	21.09	±	5.10	0.363
	NS	24.18	±	2.89	21.00	±	2.93	0.050
IM2	S	23.41	±	3.63	21.82	±	4.70	0.050
	NS	22.27	±	5.37	21.64	±	4.01	0.742
IM3	S	24.16	±	3.55	21.68	±	3.67	0.004
	NS	23.82	±	3.34	20.68	±	3.80	0.000
EM1	S	17.98	±	6.12	17.02	±	6.36	0.496
	NS	18.82	±	7.10	19.64	±	4.01	0.547
EM2	S	22.18	±	4.98	21.55	±	4.34	0.619
	NS	22.73	±	7.72	19.91	±	3.73	0.106
EM3	S	21.18	±	3.98	20.57	±	3.91	0.513
	NS	19.50	±	7.32	19.59	±	4.25	0.951
DM	S	9.98	±	5.62	12.11	±	5.97	0.117
	NS	12.09	±	5.87	14.59	±	6.63	0.224
ƩIM	S	69.55	±	10.7	64.59	±	11.9	0.022
	NS	70.27	±	10.9	63.32	±	8.54	0.050
ƩEM	S	61.34	±	12.1	59.14	±	12.0	0.430
	NS	61.05	±	21.1	59.14	±	11.1	0.613

^1^ TR: Training; CM: Competition; IM1: IM to knowledge; IM2: IM to achievement; IM3: IM to stimulating experiences; EM1: external regulation; EM2: introjected regulation; EM3: regulation identified; ƩIM: summation of all dimensions of IM; ƩEM: summation of all dimensions of EM; DM: demotivation; S: success; NS: non-success.

**Table 3:** Spearman’s rho and Pearson’s r tests between motivation and academic performance with physical characteristics.

	Motivation	MAP	LAP	EAP	GAP	Weight	FM (%)	Height
TR ^1^	IM1	−0.367 ^*^	−0.349	−0.304	−0.378 ^*^	−0.050	−0.103	0.123
	IM2	−0.195	−0.109	−0.108	−0.124	0.061	0.021	0.333 ^*^
	IM3	−0.147	−0.183	−0.041	−0.082	−0.027	−0.114	0.033
	EM1	−0.547 ^**^	−0.506 ^**^	−0.432 ^*^	−0.497 ^**^	0.057	0.066	0.124
	EM2	−0.383 ^*^	−0.266	−0.210	−0.341	0.109	0.135	0.152
	EM3	−0.309	−0.370 ^*^	−0.197	−0.283	0.211	0.114	0.240
	DM	−0.323	−0.205	−0.132	−0.235	0.045	0.099	−0.115
	ƩIM	−0.280	−0.266	−0.209	−0.261	0.012	−0.100	0.192
	ƩEM	−0.486 ^**^	−0.454 ^*^	−0.316	−0.436 ^*^	0.185	0.156	0.251
CM	IM1	−0.240	−0.050	−0.085	−0.210	0.096	0.005	0.107
	IM2	−0.058	0.098	0.070	0.020	0.458 ^**^	0.160	0.433 ^**^
	IM3	0.077	0.143	0.199	0.172	0.109	−0.060	0.350 ^*^
	EM1	−0.144	−0.206	−0.048	−0.198	0.145	0.118	0.020
	EM2	0.145	0.101	0.012	0.069	0.156	0.132	0.416 ^*^
	EM3	−0.306	−0.234	−0.230	−0.284	0.266	0.016	0.208
	DM	−0.177	−0.627 ^**^	−0.379 ^*^	−0.451^*^	0.139	0.355 ^*^	0.027
	ƩIM	−0.076	0.068	0.071	0.001	0.255	0.034	0.331
	ƩEM	−0.155	−0.209	−0.096	−0.218	0.233	0.117	0.188

^1^ TR: training; CM: competition; * *p* < 0.05; ** *p* < 0.01; IM1: IM to knowledge; IM2: IM to achievement; IM3: IM to stimulating experiences; EM1: external regulation; EM2: introjected regulation; EM3: regulation identified; DM: demotivation. MAP: math academic performance; LAP: language academic performance; EAP: English academic performance; GAP: general academic performance; FM (%): fat mass in percentage; TRn: Training week number.
